# Advancing Continuous Distribution Generation: An Exponentiated Odds Ratio Generator Approach

**DOI:** 10.3390/e26121006

**Published:** 2024-11-22

**Authors:** Xinyu Chen, Zhenyu Shi, Yuanqi Xie, Zichen Zhang, Achraf Cohen, Shusen Pu

**Affiliations:** 1 Department of Mathematics and Statistics, University of West Florida, Pensacola, FL 32514, USA; xinyu.chen3@mail.mcgill.ca (X.C.); acohen@uwf.edu (A.C.); 2 Department of Epidemiology, Biostatistics and Occupational Health, McGill University, Montreal, QC H3A 0C7, Canada; 3 Department of Communications Engineering, Zhejiang University of Science and Technology, Hangzhou 310018, China; 15906572525@163.com; 4 Department of Computer Science, University of West Florida, Pensacola, FL 32514, USA; yxie@uwf.edu; 5 Department of Mathematical and Computational Sciences, University of Toronto Mississauga, Mississauga, ON L5L 1C6, Canada; ziichen.zhang@mail.utoronto.ca

**Keywords:** continuous statistical distribution generator, exponentiated odds ratio, survival analysis, methods of estimation, statistical properties, 62E99, 60E05

## Abstract

This paper presents a new methodology for generating continuous statistical distributions, integrating the exponentiated odds ratio within the framework of survival analysis. This new method enhances the flexibility and adaptability of distribution models to effectively address the complexities inherent in contemporary datasets. The core of this advancement is illustrated by introducing a particular subfamily, the “Type 2 Gumbel Weibull-G family of distributions”. We provide a comprehensive analysis of the mathematical properties of these distributions, including statistical properties such as density functions, moments, hazard rate and quantile functions, Rényi entropy, order statistics, and the concept of stochastic ordering. To test the robustness of our new model, we apply five distinct methods for parameter estimation. The practical applicability of the Type 2 Gumbel Weibull-G distributions is further supported through the analysis of three real-world datasets. These real-life applications illustrate the exceptional statistical precision of our distributions compared to existing models, thereby reinforcing their significant value in both theoretical and practical statistical applications.

## 1. Introduction

With the ever-increasing complexity and volume of data across various disciplines, developing new statistical distributions has become a paramount area of research. These continuous distributions are essential in modeling, forecasting, and interpreting complex data, facilitating hidden patterns and relationships [1]. Nonetheless, the rapid growth and evolving nature of contemporary data often pose challenges that traditional distributions struggle to cope with, leading to a need for novel statistical distributions [2].

Traditional statistical models often face significant challenges when tasked with generating bathtub-shaped density and hazard rate functions. The concept of the bathtub curve originated in reliability engineering, where it was used to describe the life cycles of mechanical and electronic components. The initial phase of high failure rates, known as infant mortality, is often due to manufacturing defects or initial quality issues. This is followed by a middle phase characterized by a relatively constant failure rate, representing the useful life of the product. The final phase, marked by an increasing failure rate, corresponds to the wear-out period where aging and deterioration dominate [3]. Classical models like the exponential, gamma, and Weibull distributions typically show a monotonic hazard function. This limits their ability to account for the complexities inherent in bathtub-shaped data. The non-monotonic nature of these functions, which involves decreasing hazard rates after an initial peak and increasing rates as the system ages, poses a significant modeling challenge. The inadequacies of these traditional models necessitate the development of more sophisticated statistical techniques to capture the true dynamics of the underlying processes [4].

Over the past several decades, numerous methods for creating new continuous probability distributions have been explored. These include methods such as transformations of random variables, the use of mixed models, and advanced compounding methods [5,6,7,8]. Among the recent contributions in this field are the gamma–Topp–Leone–Type II–exponentiated half logistic-G distribution family [9], an enhanced version of the generalized Weibull distribution [10], the innovative modified alpha power Weibull-X distribution set [11], the Topp–Leone type II exponentiated half logistic-G distribution category [12], the modified-half-normal distribution by ref. [13], an inventive extension of the power Lindley distribution [13], the truncated inverse generalized Rayleigh distribution [14], the Ristić–Balakrishnan–Topp–Leone–Gompertz-G family of distributions [15], the odd Gompertz-G distribution family [16], the inverse Burr–generalized distribution series [17], the gamma inverse paralogistic distribution [18], and the shifted generalized truncated Nadarajah–Haghighi distribution by ref. [19].

Traditional distributions such as exponential, Weibull, gamma, and log-logistic have broad applications across various fields. However, they often struggle to model data with complex shapes, such as the bathtub curve. Recent models, such as those described in refs. [9,12,15,19], have successfully generated bathtub shapes, but the complexity of these distributions makes them difficult to comprehend and apply in practical scenarios. Additionally, statistical inference for these models can be challenging. A comprehensive review by Scheidegger et al. [20] highlights a unified perspective on various approaches to modeling such distributions, particularly in water distribution systems. Additionally, Romaniuk and Hryniewicz [21] demonstrated the utility of distributions with linear segments for capturing unique hazard rate functions, such as those encountered in pipeline maintenance cost estimation. Recent developments in reliability modeling, such as the work by Chachra et al. [22], have explored Markovian frameworks within fuzzy settings, providing further flexibility in addressing these challenges. These perspectives underscore the importance of novel methodologies, such as the one proposed in this study, to address the increasing complexity of modern data. The R-transferred exponentiated odds ratio generator introduced in this paper provides a general framework that is both concise and straightforward, as illustrated in Equation (3). This new generator is designed to offer greater flexibility and adaptability, making it well suited for managing the increasing complexity of contemporary datasets.

The foundation of this generator’s development lies in survival analysis, drawing inspiration from the extreme values of odds ratio data, as elaborated in Section 3. The generator proposed herein aims to provide a comprehensive framework for the creation of a broad range of distributions, each characterized by unique shapes and properties. This feature allows for more precise modeling and analysis of an extensive variety of data. The adaptability of the proposed generator is illustrated through its application in deriving new families of distributions, demonstrating superior data fitting capabilities compared with traditional methods.

The structure of the remaining sections of this paper is outlined as follows: Section 2 provides a description of the methodology of the new generator. Section 3 presents the new subfamily of distributions generated by the proposed technique and investigates the mathematical properties of the resulting distributions. Four estimation methods are discussed in Section 4 to test the robustness of the model. Section 5 introduces several special cases of the new family of distributions. In Section 6, we evaluate the performance and flexibility of the generator through a set of experiments and comparative analyses. Finally, we conclude the paper and discuss the future directions in Section 7.

## 2. The New Generator Based on Odds Ratio

Odds ratio quantifies the probability that an individual or component, defined by a specific lifespan following a continuous distribution H(x,ψ), will fail or expire at a particular point in time, *x* [23]. The parameters of the baseline function *H* are denoted by ψ. The odds ratio, expressed as $H(x,\psi )/\bar{H}\left(x,\psi \right)$, has become an important tool for understanding and assessing risk factors, shedding light on the relative probabilities of event outcomes, namely, death or failure [24,25,26].

This study presents a novel generator developed by integrating the methodologies delineated in [6,27]. This new generator can be efficiently applied to any baseline distribution with a cumulative distribution function (cdf), represented as R(t). It is mathematically defined by the following:

(1) $$\begin{array}{cc} F_{\mathrm{RT}\text{-}\mathrm{EOR}\text{-}H}\left(x;\alpha ,\beta \right) & =\int_{0}^{-\log(1-F_{WG}\left(x\right))}r\left(t\right)dt \end{array}$$

(2) $$\begin{array}{cc} \; & =R\left\{-\log(1-F_{WG}\left(x\right))\right\} \end{array}$$

(3) $$\begin{array}{cc} \; & =R\left\{\alpha {\left[\frac{H(x,\psi )}{\bar{H}\left(x,\psi \right)}\right]}^{\beta }\right\} \end{array}$$

Here, r(t) and R(t) denote the probability density function (pdf) and cdf of a continuous random variable, respectively. $f_{WG}\left(x\right)$, and $F_{WG}\left(x\right)$ represent the pdf and cdf of the Weibull-G family of distributions proposed by Bourguignon et al. [6]. H(x,ψ) and $\bar{H}\left(x,\psi \right)$ represent the cdf and the survival function of the baseline distribution, respectively. α and β are positive shape parameters, as defined in [6]. This newly designed generator of continuous distributions is denoted as the R-transferred exponentiated odds ratio generator (RT-EOR-H), denoted as $F_{\mathrm{RT}\text{-}\mathrm{EOR}\text{-}H}\left(x;\alpha ,\beta \right)$. While R(t) and H(x,ψ) are both cdfs, it is advisable to select a simpler format for H(x,ψ) in real-life applications. 

This new approach provides a comprehensive framework, allowing researchers to delve into the exponentiated odds ratio of any baseline function H(x,ψ), embedded within any conceivable distribution R(t). Crucially, this new generator, by permitting the investigation of any baseline function within any distribution *R*, significantly enhances the flexibility, yielding more accurate, adaptable, and precise predictions. These advancements meet the need for continued evolution in reliability analysis, providing a path for enhanced comprehension of the complexities of survival and risk.

The proposed model in this paper introduces two layers of flexibility, enabling the generation of various families of distributions. As shown in Equation (3), for any given cdf, it can be assigned as either the “outer transformer”, R(t), or the “inner driver”, H(x,ψ). This composite method significantly enhances the flexibility of possible density and hazard rate shapes, especially when using simple cdfs. 

Some example subfamilies are presented in Table 1. Additional subfamilies of the proposed model have been discussed in our recent work, including [28,29]. These subfamilies demonstrate the remarkable adaptability of the proposed model and its broad applicability to various complex datasets. When a simple “inner driver” is selected, as in the special case discussed in Section 3, the Type 2 Gumbel Weibull-G family exhibits a straightforward and elegant form. The special cases highlighted in this paper illustrate how this intuitively accessible framework can transform basic distributions, such as uniform and exponential distributions, into highly flexible shapes. Moreover, employing the same distribution for both the inner and outer components introduces intriguing behaviors, which will be addressed in detail in our future work. 

## 3. The Type 2 Gumbel Weibull-G Family of Distributions

To illustrate the applicability of the new generator, we will focus on one of its subfamilies in this paper. Here, we combine the generator with the cdf of Type 2 Gumbel distribution as follows:

(4) $$\begin{array}{c} R\left(t\right)=e^{-\lambda t^{-\delta }}, \end{array}$$

where λ and δ are real numbers and λ is the shape parameter. Then, we obtain the cdf and pdf of a new Type 2 Gumbel Weibull-G (T2GWG) family of distributions as

(5) $$\begin{array}{c} F_{T2GWG}\left(x\right)=\exp\left\{-\lambda \alpha^{-\delta }{\left[\frac{H(x,\psi )}{\bar{H}\left(x,\psi \right)}\right]}^{-\beta \delta }\right\} \end{array}$$

To avoid over-parameterization, we let λ=1, δ=1, and substitute $\alpha =\alpha^{-1}$; now, the cdf reduces to

(6) $$\begin{array}{cc} F_{T2GWG}\left(x\right) & =\exp\left\{-\alpha {\left[\frac{H(x,\psi )}{\bar{H}\left(x,\psi \right)}\right]}^{-\beta }\right\} \end{array}$$

and

(7) $$\begin{array}{c} f_{T2GWG}\left(x\right)=\alpha \beta h\left(x,\psi \right)\frac{H{\left(x,\psi \right)}^{-\beta -1}}{\bar{H}{\left(x,\psi \right)}^{-\beta +1}}\exp\left\{-\alpha {\left[\frac{H(x,\psi )}{\bar{H}\left(x,\psi \right)}\right]}^{-\beta }\right\} \end{array}$$

The T2GWG distribution emerges as a notable variant within the Odd Inverse Weibull-G family [30], distinguished by setting λ=1, and serves as an expansion to the Odd Fréchet-G framework [31]. The exploration of this particular distribution’s statistical attributes remains relatively uncharted in the existing literature. Because of its straightforwardness and minimal parameter requirements, we delve into the intricacies of the T2GWG distribution as a prime exemplar of the RT-EOR-H generator’s potential. This initial exploration sets the stage for a more comprehensive examination of additional models in forthcoming studies.

An interpretation of the T2GWG family of distributions can be given as follows. Let *Z* be a lifetime random variable with a baseline cdf H(x,ψ). The odds ratio that an individual following the lifetime *Z* will die (failure) at time *x* is $\frac{H(x,\psi )}{\bar{H}\left(x,\psi \right)}$. For a sequence of such independent and identical odds ratios $Z_{1},Z_{2},\cdots ,Z_{n}$, maximum $M_{n}=\max\left(Z_{1},Z_{2},\cdots ,Z_{n}\right)$, $M_{n}$ follows a distribution which converges to the T2GWG distribution as provided in Equation (6) [5,6].

In the subsequent subsections, we delve into the statistical properties of this novel distribution. Our discussion will cover a broad range of topics, including the expansion of the density function, hazard rate and quantile functions, moments, incomplete moments, the generation function, Rényi entropy, order statistics, and stochastic ordering.

### 3.1. Expansion of the pdf

Consider the following expansion

(8) $$\begin{array}{c} \exp\left\{-\alpha {\left[\frac{H(x,\psi )}{\bar{H}\left(x,\psi \right)}\right]}^{-\beta }\right\}=\sum_{j=0}^{\infty }\frac{{\left(-1\right)}^{j}\alpha^{j}}{j!}{\left[\frac{H(x,\psi )}{\bar{H}\left(x,\psi \right)}\right]}^{-j\beta }, \end{array}$$

then the pdf of T2GWG can be expanded as

(9) $$\begin{array}{cc} f_{T2GWG}\left(x\right) & =\alpha \beta h\left(x,\psi \right)\frac{H{\left(x,\psi \right)}^{-\beta -1}}{\bar{H}{\left(x,\psi \right)}^{-\beta +1}}\sum_{j=0}^{\infty }\frac{{\left(-1\right)}^{j}\alpha^{j}}{j!}{\left[\frac{H(x,\psi )}{\bar{H}\left(x,\psi \right)}\right]}^{-j\beta }\\ \; & =\alpha \beta h\left(x,\psi \right)\sum_{j=0}^{\infty }\frac{{\left(-1\right)}^{j}\alpha^{j}}{j!}\frac{\bar{H}{\left(x,\psi \right)}^{\beta +j\beta -1}}{H{\left(x,\psi \right)}^{\beta +j\beta +1}} \end{array}$$

Moreover, note that

(10) H¯(x,ψ)β+jβ−1=[1−H(x,ψ)]β+jβ−1=∑k=0∞β(j+1)−1k(−1)kH(x,ψ)k

Then,

(11) fT2GWG(x)=αβh(x,ψ)∑j=0∞(−1)jαjj!∑k=0∞β(j+1)−1k(−1)kH(x,ψ)kH(x,ψ)β+jβ+1=αβh(x,ψ)∑j,k=0∞(−1)j+kαjj!β(j+1)−1kH(x,ψ)k−β−jβ−1=∑j,k=0∞cj,krk−β(j+1)−1(x,ψ)

where

(12) cj,k=αβ(−1)j+kαjj!(k−β(j+1))β(j+1)−1k

and

$$r_{k-\beta -j\beta -1}\left(x,\psi \right)=\left(k-\beta \left(j+1\right)\right)h\left(x,\psi \right)H{\left(x,\psi \right)}^{k-\beta (j+1)-1}$$

which is the pdf of the exponentiated generalized (EG) distribution with parameter $\beta^{*}=k-\beta \left(j+1\right)$.

### 3.2. Hazard Rate and Quantile Functions

Building upon our earlier discussion on the odds ratio and survival analysis, we now delve into the key mathematical structures: the hazard rate function and the quantile function. These are crucial to the deeper understanding and application of survival analysis as they allow the computation of survival probabilities and survival times.

#### 3.2.1. Hazard Rate and Quantile Functions

The hazard rate function (hrf) plays an important role in survival analysis as it defines the instantaneous potential per unit time for the occurrence of an event given survival up to that time. On the other hand, the quantile function is essential in determining the time at which a certain proportion of survival is expected. In the newly proposed family of distributions, these functions take a particularly interesting form as follows.

(13) $$\begin{array}{c} h_{T2GWG}\left(x\right)=\frac{\alpha \beta h\left(x,\psi \right)\frac{H{\left(x,\psi \right)}^{-\beta -1}}{\bar{H}{\left(x,\psi \right)}^{-\beta +1}}\exp\left\{-\alpha {\left[\frac{H(x,\psi )}{\bar{H}\left(x,\psi \right)}\right]}^{-\beta }\right\}}{1-\exp\left\{-\alpha {\left[\frac{H(x,\psi )}{\bar{H}\left(x,\psi \right)}\right]}^{-\beta }\right\}} \end{array}$$

In addition to the hrf, it is also useful to consider its reciprocal, termed the reverse hazard rate function. This function essentially reflects the hazard function’s properties but is viewed from the perspective of the event not occurring.

(14) $$\begin{array}{cc} \tau_{T2GWG}\left(x\right) & =\frac{\alpha \beta h\left(x,\psi \right)\frac{H{\left(x,\psi \right)}^{-\beta -1}}{\bar{H}{\left(x,\psi \right)}^{-\beta +1}}\exp\left\{-\alpha {\left[\frac{H(x,\psi )}{\bar{H}\left(x,\psi \right)}\right]}^{-\beta }\right\}}{\exp\left\{-\alpha {\left[\frac{H(x,\psi )}{\bar{H}\left(x,\psi \right)}\right]}^{-\beta }\right\}}\\ \; & =\alpha \beta h\left(x,\psi \right)\frac{H{\left(x,\psi \right)}^{-\beta -1}}{\bar{H}{\left(x,\psi \right)}^{-\beta +1}} \end{array}$$

#### 3.2.2. Quantile Function

Next, we will focus on the quantile function, which is particularly useful when determining the survival time corresponding to a specific survival probability.

$$F_{T2GWG}\left(x\right)=\exp\left\{-\alpha {\left[\frac{H(x,\psi )}{\bar{H}\left(x,\psi \right)}\right]}^{-\beta }\right\}=p$$

For 0≤p≤1, then, it is sufficient to solve

(15) $$\begin{array}{c} H\left(x,\psi \right)=\frac{1}{{\left(\frac{\log p}{-\alpha }\right)}^{\frac{1}{\beta }}+1}:=q \end{array}$$

Thus, the quantile $x_{p}$ of the distribution reduces to the quantile $x_{q}$ of the baseline distribution with cdf H(x,ψ) and is given by

(16) $$\begin{array}{c} x_{q}=H^{-1}\left(q\right) \end{array}$$

### 3.3. Moments, Incomplete Moments, and Generating Functions

#### 3.3.1. Moments

In the field of statistics, moments play a vital role in characterizing the properties of a probability distribution. Moments provide important summary measures of the characteristics of datasets. The first moment about the origin, also known as the mean, measures the location of the distribution. The second central moment is known as the variance, which quantifies the spread or dispersion of the distribution. The third and fourth moments, skewness and kurtosis, respectively, describe the shape of the distribution, capturing aspects of its asymmetry and tailedness. We can present the $r^{th}$ moment of the distributions as

(17) $$\begin{array}{c} E\left(Y^{r}\right)=\sum_{j,k=0}^{\infty }c_{j,k}E\left(Z_{j,k}^{r}\right) \end{array}$$

where $Z_{j,k}$ is the exponentiated generalized distribution with the parameter $\beta^{*}=k-\beta \left(j+1\right)$ and $c_{j,k}$ is defined by Equation (12).

#### 3.3.2. Incomplete Moments, Conditional Moments, and Moment Generating Function

While moments give us an understanding of the general characteristics of a distribution, they do not always provide sufficient detail about specific intervals or subsets within the data. This is where incomplete moments come into play. Incomplete moments, also known as truncated or restricted moments, are defined similarly to regular moments but are integrated over a subset of the possible range of the variable. They offer a more granular insight into the characteristics of the distribution within specific ranges. This makes them particularly useful when analyzing left- or right-skewed data, or when assessing the impact of outlier observations. The incomplete moment is provided as

(18) $$\begin{array}{c} I_{Y}\left(z\right)=\int_{0}^{z}y^{s}f_{T2GWG}\left(y\right)dy=\sum_{j,k=0}^{\infty }c_{j,k}I_{j,k}\left(y\right) \end{array}$$

where $I_{j,k}\left(y\right)=\int_{0}^{z}y^{s}r_{k-\beta (j+1)}\left(x,\psi \right)$.

The rth conditional moments of the Type 2 Gumbel Weibull-G family of distributions is given by

(19) $$\begin{array}{cc} E(Y^{r}|Y\geq a) & =\frac{1}{{\bar{F}}_{T2GWG}\left(a;\alpha ,\beta ,\psi \right)}\int_{t}^{\infty }y^{r}f_{T2GWG}\left(y;\alpha ,\beta ,\psi \right)dy\\ \; & =\frac{1}{{\bar{F}}_{T2GWG}\left(a;\alpha ,\beta ,\psi \right)}\sum_{j,k=0}^{\infty }c_{j,k}I_{j,k}\left(y\right) \end{array}$$

where $I_{j,k}\left(y\right)$ is defined above.

The moment generating function is given by

(20) $$\begin{array}{c} M_{Y}\left(t\right)=E\left(e^{tY}\right)=\sum_{j,k=0}^{\infty }c_{j,k}E\left(e^{tZ_{j,k}}\right)=\sum_{j,k=0}^{\infty }c_{j,k}M_{Z_{j,k}}\left(t\right) \end{array}$$

and the characteristic function is defined as

(21) $$\begin{array}{c} \phi \left(t\right)=E\left(e^{itY}\right)=\sum_{j,k=0}^{\infty }c_{j,k}E\left(e^{itZ_{j,k}}\right)=\sum_{j,k=0}^{\infty }c_{j,k}\phi_{k-\beta (j+1)}\left(t\right), \end{array}$$

where $\phi_{k-\beta (j+1)}\left(t\right)$ is the characteristic function of the EG distribution with parameter $\beta^{*}=k-\beta \left(j+1\right)$.

### 3.4. Rényi Entropy

Rényi entropy is named after the Hungarian mathematician Alfréd Rényi [32]. While Shannon entropy is perhaps the most commonly referenced form of entropy in the field of information theory, characterizing the average uncertainty or unpredictability of a source of information, Rényi entropy provides a more generalized measure. Rényi entropy finds applications in various domains including physics, computer science, statistics, and quantum information theory. For example, in the context of machine learning, it can be used to measure the diversity or complexity of learned models. In statistical physics, it is useful in understanding the thermodynamics of complex systems. The Rényi entropy of this new distribution is

$$\begin{array}{cc} I_{R}\left(\omega \right)= & {\left(1-\omega \right)}^{-1}\log\left[\int_{-\infty }^{\infty }f^{\omega }\left(x\right)dx\right]\\ = & {\left(1-\omega \right)}^{-1}\left\{\omega (\log\alpha +\log\beta )\right.\\ \; & +\left.\log\left[\int_{-\infty }^{\infty }h^{\omega }\left(x,\psi \right)\frac{H{\left(x,\psi \right)}^{\omega (-\beta -1)}}{\bar{H}{\left(x,\psi \right)}^{\omega (-\beta +1)}}\exp\left\{-\omega \alpha {\left[\frac{H(x,\psi )}{\bar{H}\left(x,\psi \right)}\right]}^{-\beta }\right\}\right]\right\} \end{array}$$

where ω>0 and ω≠1. Applying the same expansion technique for the pdf, we obtain

(22) $$\begin{array}{cc} I_{R}\left(\omega \right) & ={\left(1-\omega \right)}^{-1}\left\{\omega (\log\alpha +\log\beta )\right.\\ \; & +\left.\log\left[\sum_{i=0}^{\infty }\frac{{\left(-1\right)}^{i}{\left(\omega \alpha \right)}^{i}}{i!}\int_{-\infty }^{\infty }h^{\omega }\left(x,\psi \right)\frac{{\left[\bar{H}\left(x,\psi \right)\right]}^{\omega (\beta -1)+i\beta }}{{\left[H\left(x,\psi \right)\right]}^{\omega (\beta +1)+i\beta }}dx\right]\right\} \end{array}$$

Consider that

(23) H¯(x,ψ)ω(β−1)+iβ=[1−H(x,ψ)]ω(β−1)+iβ=∑j=0∞ω(β−1)+iβj(−1)jH(x,ψ)j

Thus, we can write the Reńyi entropy as

(24) IR(ω)=(1−ω)−1ω(logα+logβ)+log∑i=0∞∑j=0∞(−1)i+j(ωα)ii!×ω(β−1)+iβj∫−∞∞hω(x,ψ)(H(x,ψ))j−ω(β+1)−iβdx=(1−ω)−1ω(logα+logβ)+log∑i=0∞∑j=0∞(−1)i+j(ωα)ii!×ω(β−1)+iβjωω[j−ω(β+1)−iβ+ω]ω×∫−∞∞[j−ω(β+1)−iβ+ωωh(x,ψ)(H(x,ψ))j−ω(β+1)−iβω]ω=(1−ω)−1ω(logα+logβ)+log∑i=0∞∑j=0∞(−1)i+j(ωα)ii!×ω(β−1)+iβjωω[j−ω(β+1)−iβ+ω]ω×e(1−ω)IREG

where $I_{REG}$ is the Rényi entropy of the exponentiated generalized distribution with parameter $\beta^{*}=\frac{j-\omega (\beta +1)-i\beta +\omega }{\omega }$.

### 3.5. Order Statistics

In the field of statistics, order statistics are a fundamental concept that allows for deeper analysis and understanding of sampled data. Specifically, order statistics are the values from a random sample sorted in ascending or descending order. This sorting process provides a powerful perspective on the sample’s overall distribution and associated characteristics. Order statistics are used in a variety of applications, including non-parametric statistics (which does not rely on parameters defined in terms of a theoretical or assumed population), reliability engineering, and statistical quality control. They also play a central role in the construction of quantile–quantile plots, which are used to assess whether a dataset follows a particular theoretical distribution.

Let $X_{1},X_{2},...,X_{n}$ be independent identically distributed random variables distributed by Equation (7). The pdf of the $i^{th}$ order statistic $f_{i:n}\left(x\right)$ is given by

(25) fi:n(x)=n!fT2GWG(x)(i−1)!(n−i)![FT2GWG(x)]i−1[1−FT2GWG(x)]n−i=n!fT2GWG(x)(i−1)!(n−i)!∑m=0n−in−im(−1)m[FT2GWG(x)]i−1+m=n!fT2GWG(x)(i−1)!(n−i)!∑m=0n−in−im(−1)mexp(i−1+m)(−α)H(x,ψ)H¯(x,ψ)−β=n!(i−1)!(n−i)!∑m=0n−in−im(−1)mi+mfT2GWG(x;(i+m)α,β)

Therefore, we can present $f_{i:n}\left(x\right)$ as a linear combination of the T2GWG with parameter $\left(\alpha^{*},\beta \right)$, where $\alpha^{*}=\left(i+m\right)\alpha$.

### 3.6. Stochastic Ordering

Stochastic ordering is a mathematical concept frequently applied in the realm of statistics, probability theory, decision theory, and economics [33]. The most basic form of stochastic ordering is the usual order of real numbers, which extends naturally to random variables: a random variable *X* is said to be stochastically smaller than another random variable *Y* if, for every real number *x*, the probability that *X* is less than or equal to *x* is higher than or equal to the probability that *Y* is less than or equal to *x*. This gives rise to the concept of one distribution being “stochastically larger” than another, which can be a valuable tool in comparing different probability models or assessing risk. There are several types of stochastic orderings, such as increasing convex order, likelihood ratio order, and hazard rate order, each imposing a different structure on the sets of random variables or distributions. Stochastic ordering is a significant concept because it enables us to make statements about the relative behavior of different random variables or distributions without specifying them precisely. It has been widely used in various fields, such as reliability, insurance, finance, operations research, and queuing theory.

Let $X_{1}∼T2GWG\left(x;\alpha_{1},\beta ,\psi \right)$ and $X_{2}∼T2GWG\left(x;\alpha_{2},\beta ,\psi \right)$. The likelihood ratio is

(26) $$\begin{array}{cc} \frac{f_{X_{1}}\left(x\right)}{f_{X_{2}}\left(x\right)} & =\frac{\alpha_{1}\beta h\left(x,\psi \right)\frac{H{\left(x,\psi \right)}^{-\beta -1}}{\bar{H}{\left(x,\psi \right)}^{-\beta +1}}\exp\left\{-\alpha_{1}{\left[\frac{H(x,\psi )}{\bar{H}\left(x,\psi \right)}\right]}^{-\beta }\right\}}{\alpha_{2}\beta h\left(x,\psi \right)\frac{H{\left(x,\psi \right)}^{-\beta -1}}{\bar{H}{\left(x,\psi \right)}^{-\beta +1}}\exp\left\{-\alpha_{2}{\left[\frac{H(x,\psi )}{\bar{H}\left(x,\psi \right)}\right]}^{-\beta }\right\}}\\ \; & =\frac{\alpha_{1}}{\alpha_{2}}\exp\left\{\left(\alpha_{2}-\alpha_{1}\right){\left[\frac{H(x,\psi )}{\bar{H}\left(x,\psi \right)}\right]}^{-\beta }\right\} \end{array}$$

Then, we differentiate Equation (26) and obtain

(27) $$\begin{array}{cc} \frac{d}{dx}\left(\frac{f_{X_{1}}\left(x\right)}{f_{X_{2}}\left(x\right)}\right) & =\frac{\alpha_{1}}{\alpha_{2}}\exp\left\{\left(\alpha_{2}-\alpha_{1}\right){\left[\frac{H(x,\psi )}{\bar{H}\left(x,\psi \right)}\right]}^{-\beta }\right\}\\ \; & \times \left[\left(\alpha_{2}-\alpha_{1}\right)h\left(x,\psi \right)\frac{H{\left(x,\psi \right)}^{-\beta -1}}{\bar{H}{\left(x,\psi \right)}^{-\beta +1}}\right] \end{array}$$

If $\alpha_{1}<\alpha_{2}$, $\frac{d}{dx}\left(\frac{f_{X_{1}}\left(x\right)}{f_{X_{2}}\left(x\right)}\right)<0$. Thus, it indicates that $X{⪯}_{lr}Y$. According to the theorem proposed by ref. [33], both $X{⪯}_{hr}Y$ and X⪯Y hold.

## 4. Methods of Estimation

### 4.1. Maximum Likelihood Estimation

We can estimate the unknown parameters of the Type 2 Gumbel Weibull-G family distributions by using the widely used maximum likelihood estimation (MLE). Let $\Delta ={\left(\alpha ,\beta ,\psi \right)}^{T}$. Then, the log-likelihood for Δ is defined by

(28) $$\begin{array}{cc} \ell (\Delta ) & =n\log\left(\alpha \right)+n\log\left(\beta \right)+\sum_{i=1}^{n}\log h\left(x_{i},\psi \right)-\left(\beta +1\right)\sum_{i=1}^{n}\log H\left(x_{i},\psi \right)\\ \; & +\left(\beta -1\right)\sum_{i=1}^{n}\log\left[1-H\left(x_{i},\psi \right)\right]-\alpha \sum_{i=1}^{n}{\left[\frac{H(x_{i},\psi )}{1-H(x_{i},\psi )}\right]}^{-\beta } \end{array}$$

The first derivatives of ℓ(Δ) with respect to Δ are shown as follows:

(29) $$\begin{array}{c} \frac{\partial \ell }{\partial \alpha }=\frac{n}{\alpha }-\sum_{i=1}^{n}{\left[\frac{H(x_{i},\psi )}{1-H(x_{i},\psi )}\right]}^{-\beta } \end{array}$$

(30) $$\begin{array}{cc} \frac{\partial \ell }{\partial \beta }= & \frac{n}{\beta }-\sum_{i=1}^{n}\log H\left(x_{i},\psi \right)+\sum_{i=1}^{n}\log\left[1-H\left(x_{i},\psi \right)\right]\\ \; & +\alpha \sum_{i=1}^{n}{\left[\frac{H(x_{i},\psi )}{1-H(x_{i},\psi )}\right]}^{-\beta }\log\left(\frac{H(x_{i},\psi )}{1-H(x_{i},\psi )}\right) \end{array}$$

and

(31) $$\begin{array}{cc} \frac{\partial \ell }{\partial \psi_{k}}= & \sum_{i=1}^{n}\frac{1}{h(x_{i},\psi )}\frac{\partial h(x_{i},\psi )}{\partial \psi_{k}}-\left(\beta +1\right)\sum_{i=1}^{n}\frac{1}{H(x_{i},\psi )}\frac{\partial H(x_{i},\psi )}{\partial \psi_{k}}\\ \; & -\left(\beta -1\right)\sum_{i=1}^{n}\frac{1}{1-H(x_{i},\psi )}\frac{\partial H(x_{i},\psi )}{\partial \psi_{k}}\\ \; & +\alpha \beta \sum_{i=1}^{n}\frac{H{\left(x_{i},\psi \right)}^{-\beta -1}}{{\left[1-H\left(x_{i},\psi \right)\right]}^{-\beta +1}}\frac{\partial H(x_{i},\psi )}{\partial \psi_{k}} \end{array}$$

where $\psi_{k}$ is the $k^{th}$ element of the vector ψ.

We can maximize the log-likelihood function ℓ(Δ) by solving the nonlinear equations $\left(\frac{\partial \ell }{\partial \alpha },\frac{\partial \ell }{\partial \beta },\frac{\partial \ell }{\partial \psi_{k}}\right)=0$ with numerical methods such as the Newton–Raphson approach.

### 4.2. Least Square and Weighted Least Square Estimation

The least square (LS) method is a commonly used technique in regression analysis for approximating the solution of overdetermined systems. The method provides the best linear unbiased estimates of the unknown parameters if the errors are homoscedastic and uncorrelated. On the other hand, the weighted least square (WLSE) approach extends the least square technique by incorporating the different variances of the observations. This method assigns a weight to each data point based on the variance of its error term, placing less emphasis on the observations with higher variances to make the overall model more reliable.

The LSE and WLSE techniques can also provide estimators in the model. The LS estimation is given by

(32) $$\begin{array}{cc} LS(\Delta ) & =\sum_{i=1}^{n}{\left(F\left(x_{i},\Delta \right)-\frac{i}{n+1}\right)}^{2}\\ \; & =\sum_{i=1}^{n}{\left(\exp\left\{-\alpha {\left[\frac{H(x_{i},\psi )}{\bar{H}\left(x_{i},\psi \right)}\right]}^{-\beta }\right\}-\frac{i}{n+1}\right)}^{2} \end{array}$$

By differentiating Equation (32), we have the following:

(33) $$\begin{array}{cc} \frac{\partial LS}{\partial \alpha }= & 2\sum_{i=1}^{n}\left(\exp\left\{-\alpha {\left[\frac{H(x_{i},\psi )}{\bar{H}\left(x_{i},\psi \right)}\right]}^{-\beta }\right\}-\frac{i}{n+1}\right)\\ \; & \times \left(-1\right){\left[\frac{H(x_{i},\psi )}{\bar{H}\left(x_{i},\psi \right)}\right]}^{-\beta }\exp\left\{-\alpha {\left[\frac{H(x_{i},\psi )}{\bar{H}\left(x_{i},\psi \right)}\right]}^{-\beta }\right\} \end{array}$$

(34) $$\begin{array}{cc} \frac{\partial LS}{\partial \beta }= & 2\sum_{i=1}^{n}\left(\exp\left\{-\alpha {\left[\frac{H(x_{i},\psi )}{\bar{H}\left(x_{i},\psi \right)}\right]}^{-\beta }\right\}-\frac{i}{n+1}\right)\\ \; & \times \alpha \log\left(\frac{H(x_{i},\psi )}{\bar{H}\left(x_{i},\psi \right)}\right){\left[\frac{H(x_{i},\psi )}{\bar{H}\left(x_{i},\psi \right)}\right]}^{-\beta }\exp\left\{-\alpha {\left[\frac{H(x_{i},\psi )}{\bar{H}\left(x_{i},\psi \right)}\right]}^{-\beta }\right\} \end{array}$$

and

(35) $$\begin{array}{cc} \frac{\partial LS}{\partial \psi_{k}}= & 2\sum_{i=1}^{n}\left(\exp\left\{-\alpha {\left[\frac{H(x_{i},\psi )}{\bar{H}\left(x_{i},\psi \right)}\right]}^{-\beta }\right\}-\frac{i}{n+1}\right)\\ \; & \times \alpha \beta \frac{\partial H(x_{i},\psi )}{\partial \psi_{k}}\frac{H{\left(x_{i},\psi \right)}^{-\beta -1}}{\bar{H}{\left(x_{i},\psi \right)}^{-\beta +1}}\exp\left\{-\alpha {\left[\frac{H(x_{i},\psi )}{\bar{H}\left(x_{i},\psi \right)}\right]}^{-\beta }\right\} \end{array}$$

We can also apply the Newton–Raphson procedure to minimize the least square estimation LS(Δ) by solving the equations $\left(\frac{\partial LS}{\partial \alpha },\frac{\partial LS}{\partial \beta },\frac{\partial LS}{\partial \theta_{k}}\right)=0$.

Similarly, the WLS estimation can be obtained by minimizing

(36) $$\begin{array}{cc} WLS(\Delta ) & =\sum_{i=1}^{n}\frac{{\left(n+1\right)}^{2}\left(n+2\right)}{i(n-i+1)}{\left(F\left(x_{i},\Delta \right)-\frac{i}{n+1}\right)}^{2}\\ \; & =\sum_{i=1}^{n}\frac{{\left(n+1\right)}^{2}\left(n+2\right)}{i(n-i+1)}{\left(\exp\left\{-\alpha {\left[\frac{H(x_{i},\psi )}{\bar{H}\left(x_{i},\psi \right)}\right]}^{-\beta }\right\}-\frac{i}{n+1}\right)}^{2} \end{array}$$

### 4.3. Maximum Product Spacing Approach of Estimation

The maximum product spacing (MPS) approach is particularly useful when dealing with unknown or complex distributions [34]. Unlike MLE, the MPS method does not require the explicit formulation of a likelihood function, making it a versatile and robust approach for different types of distributions. The geometric mean of the MPS spacings is given by

(37) $$\begin{array}{c} G\left(\Delta \right)={\left\{\prod_{i=1}^{n+1}D_{i}\left(x_{i},\Delta \right)\right\}}^{\frac{1}{n+1}} \end{array}$$

where

$$\begin{array}{c} D_{i}=\left\{\begin{array}{cc} & F(x_{1},\Delta ),i=0\\ & F\left(x_{i},\Delta \right)-F\left(x_{i-1},\Delta \right),i=2,3,...,n\\ & 1-F(x_{n},\Delta ),i=n+1 \end{array}\right. \end{array}$$

Thus, we can maximize

(38) $$\begin{array}{cc} G(\Delta )= & \left[\exp\left\{-\alpha {\left[\frac{H(x_{1},\psi )}{\bar{H}\left(x_{1},\psi \right)}\right]}^{\beta }\right\}\left(1-\exp\left\{-\alpha {\left[\frac{H(x_{n},\psi )}{\bar{H}\left(x_{n},\psi \right)}\right]}^{-\beta }\right\}\right)\right.\\ \; & {\left.\times \prod_{i=2}^{n}\left(\exp\left\{-\alpha {\left[\frac{H(x_{i},\psi )}{\bar{H}\left(x_{i},\psi \right)}\right]}^{-\beta }\right\}-\exp\left\{-\alpha {\left[\frac{H(x_{i-1},\psi )}{\bar{H}\left(x_{i-1},\psi \right)}\right]}^{-\beta }\right\}\right)\right]}^{\frac{1}{n+1}} \end{array}$$

Equivalently, we can also maximize W=logG

(39) $$\begin{array}{cc} W(\Delta )= & \frac{1}{n+1}\sum_{i=1}^{n+1}\log D_{i}\left(x_{i},\Delta \right)\\ = & \frac{1}{n+1}\left\{-\alpha {\left[\frac{H(x_{1},\psi )}{\bar{H}\left(x_{1},\psi \right)}\right]}^{-\beta }+\log\left(1-\exp\left\{-\alpha {\left[\frac{H(x_{n},\psi )}{\bar{H}\left(x_{n},\psi \right)}\right]}^{-\beta }\right\}\right)\right.\\ \; & \left.+\sum_{i=2}^{n}\log\left(\exp\left\{-\alpha {\left[\frac{H(x_{i},\psi )}{\bar{H}\left(x_{i},\psi \right)}\right]}^{-\beta }\right\}-\exp\left\{-\alpha {\left[\frac{H(x_{i-1},\psi )}{\bar{H}\left(x_{i-1},\psi \right)}\right]}^{-\beta }\right\}\right)\right\} \end{array}$$

By solving $\left(\frac{\partial W}{\partial \alpha },\frac{\partial W}{\partial \beta },\frac{\partial W}{\partial \psi_{k}}\right)=0$, we can obtain the estimators. The partial derivatives are provided in Appendix A.

### 4.4. Cramér–Von Mises Approach of Estimation

The Cramér–von Mises method is another approach to estimate the parameters of a distribution [35]. The Cramér–von Mises statistic measures the difference between the empirical distribution function of the data and the cumulative distribution function of the proposed model. This technique has an advantage over methods such as maximum likelihood estimation in that it considers the whole dataset, not just the location and dispersion, resulting in a more comprehensive estimation. We can apply the Cramér–von Mises criterion to obtain the estimators by minimizing the function S(x;Δ) with respect to Δ, where

(40) $$\begin{array}{cc} S(x;\Delta ) & =\frac{1}{12n^{2}}+\frac{1}{n}\sum_{i=1}^{n}{\left(F\left(x_{i};\Delta \right)-\frac{2i-1}{2n}\right)}^{2}\\ \; & =\frac{1}{12n^{2}}+\frac{1}{n}\sum_{i=1}^{n}{\left(\exp\left\{-\alpha {\left[\frac{H(x_{i},\psi )}{\bar{H}\left(x_{i},\psi \right)}\right]}^{-\beta }\right\}-\frac{2i-1}{2n}\right)}^{2} \end{array}$$

Take the first partial derivatives of S, and we can have the following:

(41) $$\begin{array}{cc} \frac{\partial S}{\partial \alpha }= & \frac{2}{n}\sum_{i=1}^{n}\left(\exp\left\{-\alpha {\left[\frac{H(x_{i},\psi )}{\bar{H}\left(x_{i},\psi \right)}\right]}^{-\beta }\right\}-\frac{2i-1}{2n}\right)\\ \; & \times \left(-1\right){\left[\frac{H(x_{i},\psi )}{\bar{H}\left(x_{i},\psi \right)}\right]}^{-\beta }\exp\left\{-\alpha {\left[\frac{H(x_{i},\psi )}{\bar{H}\left(x_{i},\psi \right)}\right]}^{-\beta }\right\} \end{array}$$

(42) $$\begin{array}{cc} \frac{\partial S}{\partial \beta }= & \frac{2}{n}\sum_{i=1}^{n}\left(\exp\left\{-\alpha {\left[\frac{H(x_{i},\psi )}{\bar{H}\left(x_{i},\psi \right)}\right]}^{-\beta }\right\}-\frac{2i-1}{2n}\right)\\ \; & \times \alpha \log\left(\frac{H(x_{i},\psi )}{\bar{H}\left(x_{i},\psi \right)}\right){\left[\frac{H(x_{i},\psi )}{\bar{H}\left(x_{i},\psi \right)}\right]}^{-\beta }\exp\left\{-\alpha {\left[\frac{H(x_{i},\psi )}{\bar{H}\left(x_{i},\psi \right)}\right]}^{-\beta }\right\} \end{array}$$

(43) $$\begin{array}{cc} \frac{\partial S}{\partial \psi_{k}}= & \frac{2}{n}\sum_{i=1}^{n}\left(\exp\left\{-\alpha {\left[\frac{H(x_{i},\psi )}{\bar{H}\left(x_{i},\psi \right)}\right]}^{-\beta }\right\}-\frac{2i-1}{2n}\right)\\ \; & \times \alpha \beta \frac{\partial H(x_{i},\psi )}{\partial \psi_{k}}\frac{H{\left(x_{i},\psi \right)}^{-\beta -1}}{\bar{H}{\left(x_{i},\psi \right)}^{-\beta +1}}\exp\left\{-\alpha {\left[\frac{H(x_{i},\psi )}{\bar{H}\left(x_{i},\psi \right)}\right]}^{-\beta }\right\} \end{array}$$

### 4.5. Anderson and Darling Approach of Estimation

The Anderson–Darling approach was proposed in ref. [36] to test whether a dataset follows a specific distribution, and can also be used to estimate parameters. This method gives greater weight to the tails of the distribution compared to other methods, like the Kolmogorov–Smirnov test. The Anderson–Darling statistic is minimized to find the parameters of the best-fitting distribution. This estimation method is highly sensitive to deviations in the tails and thus can be more powerful for identifying whether a particular distribution fits the data. The Anderson–Darling estimators can be obtained by minimizing

(44) $$\begin{array}{cc} AD(\Delta )= & -n-\frac{1}{n}\sum_{i=1}^{n}\left(2i-1\right)\left[\log F\left(x_{i},\Delta \right)-\log\bar{F}\left(x_{n+1-i},\Delta \right)\right]\\ = & -n-\frac{1}{n}\sum_{i=1}^{n}\left(2i-1\right)\left[-\alpha {\left[\frac{H(x_{i},\psi )}{\bar{H}\left(x_{i},\psi \right)}\right]}^{-\beta }\right.\\ \; & -\left.\log\left(1-\exp\left\{-\alpha {\left[\frac{H(x_{n+1-i},\psi )}{\bar{H}\left(x_{n+1-i},\psi \right)}\right]}^{-\beta }\right\}\right)\right] \end{array}$$

Similarly, we take the first derivatives of AD(Δ) and obtain

(45) $$\begin{array}{cc} \frac{\partial AD}{\partial \alpha }= & -\frac{1}{n}\sum_{i=1}^{n}\left(2i-1\right)\left(-{\left[\frac{H(x_{i},\psi )}{\bar{H}\left(x_{i},\psi \right)}\right]}^{-\beta }\right.\\ \; & -\left.{\left[\frac{H(x_{n+1-i},\psi )}{\bar{H}\left(x_{n+1-i},\psi \right)}\right]}^{-\beta }\frac{\exp\left\{-\alpha {\left[\frac{H(x_{n+1-i},\psi )}{\bar{H}\left(x_{n+1-i},\psi \right)}\right]}^{-\beta }\right\}}{1-\exp\left\{-\alpha {\left[\frac{H(x_{n+1-i},\psi )}{\bar{H}\left(x_{n+1-i},\psi \right)}\right]}^{-\beta }\right\}}\right) \end{array}$$

(46) $$\begin{array}{cc} \frac{\partial AD}{\partial \beta }= & -\frac{1}{n}\sum_{i=1}^{n}\left(2i-1\right)\left[\alpha \log\left(\frac{H(x_{i},\psi )}{\bar{H}\left(x_{i},\psi \right)}\right){\left[\frac{H(x_{i},\psi )}{\bar{H}\left(x_{i},\psi \right)}\right]}^{-\beta }\right.\\ \; & +\alpha \log\left(\frac{H(x_{n+1-i},\psi )}{\bar{H}\left(x_{n+1-i},\psi \right)}\right){\left[\frac{H(x_{n+1-i},\psi )}{\bar{H}\left(x_{n+1-i},\psi \right)}\right]}^{-\beta }\frac{\exp\left\{-\alpha {\left[\frac{H(x_{n+1-i},\psi )}{\bar{H}\left(x_{n+1-i},\psi \right)}\right]}^{-\beta }\right\}}{1-\exp\left\{-\alpha {\left[\frac{H(x_{n+1-i},\psi )}{\bar{H}\left(x_{n+1-i},\psi \right)}\right]}^{-\beta }\right\}} \end{array}$$

(47) $$\begin{array}{cc} \frac{\partial AD}{\partial \psi_{k}}= & -\frac{1}{n}\sum_{i=1}^{n}\left(2i-1\right)\left[\alpha \beta \frac{H{\left(x_{i},\psi \right)}^{-\beta -1}}{\bar{H}{\left(x_{i},\psi \right)}^{-\beta +1}}\frac{\partial H(x_{i},\psi )}{\partial \psi_{k}}\right.\\ \; & \left.+\alpha \beta \frac{H{\left(x_{n+1-i},\psi \right)}^{-\beta -1}}{\bar{H}{\left(x_{n+1-i},\psi \right)}^{-\beta +1}}\frac{\partial H(x_{n+1-i},\psi )}{\partial \psi_{k}}\frac{\exp\left\{-\alpha {\left[\frac{H(x_{n+1-i},\psi )}{\bar{H}\left(x_{n+1-i},\psi \right)}\right]}^{-\beta }\right\}}{1-\exp\left\{-\alpha {\left[\frac{H(x_{n+1-i},\psi )}{\bar{H}\left(x_{n+1-i},\psi \right)}\right]}^{-\beta }\right\}}\right] \end{array}$$

### 4.6. Simulation and Estimation

**Figure 1:**
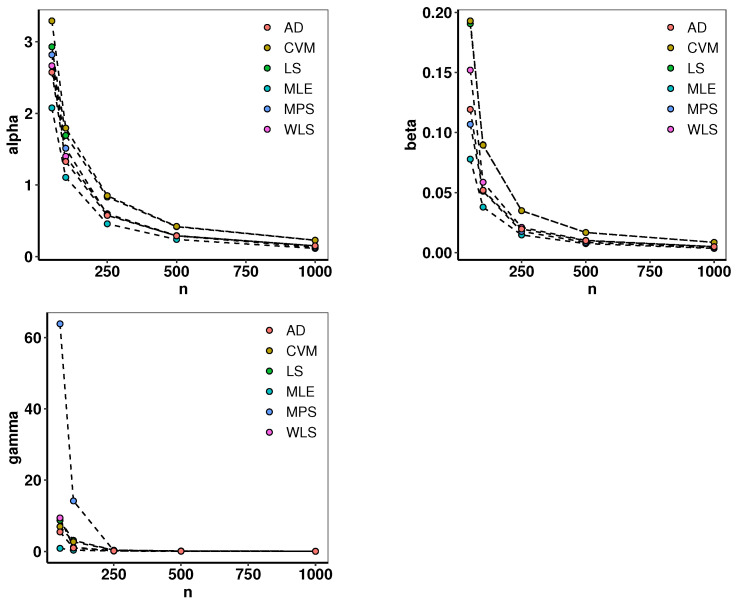
MSE of parameters in Table 3.

We combined Monte Carlo simulation with the above estimation techniques to estimate the parameters of distributions. The parameters are set as α=2.5,β=0.8, and γ=1.3. The sample sizes N=50,100,250,500, and 1000 were used to generate random samples. For each sample size, the experiment was replicated N=1000 times. Then, the bias and mean squared error (MSE) were calculated for each set of data. Detailed pseudo-code for simulating our proposed model using a Monte Carlo framework is provided in Table 2. Table 3 and Figure 1 show the estimation results. The MSE converges to 0 as *N* increases, confirming the estimations’ stability and reliability in all cases.

## 5. Special Cases

In this section, we will explore a variety of special cases that emerge from our novel distribution model. By closely examining these unique instances, we aim to illustrate the multifaceted aspects and potential applications of the T2GWG family of distributions.

### 5.1. Type 2 Gumbel Weibull–Exponential (T2GWE) Distribution

Suppose the baseline distribution H(x,ψ) is an exponential distribution with parameter γ>0. Then, $h\left(x;\gamma \right)=\gamma e^{-\gamma x}$ and $H\left(x;\gamma \right)=1-e^{-\gamma x}$.

#### 5.1.1. cdf and pdf of the T2GWE Distribution

The cdf of the Type 2 Gumbel Weibull–exponential distribution is presented as

(48) $$\begin{array}{c} F_{T2GWE}\left(x\right)=\exp\left\{-\alpha {\left(e^{\gamma x}-1\right)}^{-\beta }\right\} \end{array}$$

and the pdf is given by

(49) $$\begin{array}{c} f_{T2GWE}\left(x\right)=\beta \alpha {\left(e^{\gamma x}-1\right)}^{-\beta -1}\exp\left\{\gamma x-\alpha {\left(e^{\gamma x}-1\right)}^{-\beta }\right\} \end{array}$$

where x≥0 and α,β>0.

#### 5.1.2. Hazard Rate and Quantile Functions

The hrf is shown as

(50) $$\begin{array}{c} h_{T2GWE}=\frac{\beta \alpha {\left(e^{\gamma x}-1\right)}^{-\beta -1}\exp\left\{\gamma x-\alpha {\left(e^{\gamma x}-1\right)}^{-\beta }\right\}}{1-\exp\left\{-\alpha {\left(e^{\gamma x}-1\right)}^{-\beta }\right\}} \end{array}$$

and the reverse hrf is given by

(51) $$\begin{array}{c} \tau_{T2GWE}=\beta \alpha {\left(e^{\gamma x}-1\right)}^{-\beta -1} \end{array}$$

Moreover, the quantile function is obtained as

(52) $$\begin{array}{c} x_{p}=-\frac{1}{\gamma }\log\left({\left[\frac{\log p}{-\alpha }\right]}^{-\frac{1}{\beta }}+1\right) \end{array}$$

**Figure 2:**
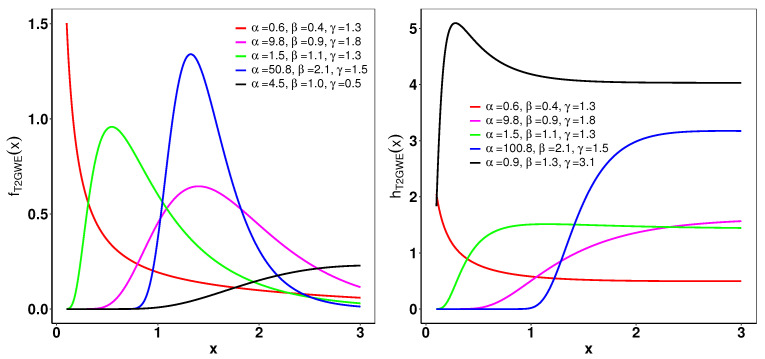
(**left**): The pdf of the T2GWE distribution for different parameters. (**right**): The hrf of the T2GWE for different parameter values.

Figure 2 displays several typical configurations of the pdf and hrf for the T2GWE distribution. The pdf of the T2GWE distribution shows various configurations, including almost symmetric, right-skewed, decreasing, and increasing. Additionally, the hrf of the T2GWE distribution can exhibit a range of shapes, such as decreasing, increasing, and right-skewed.

### 5.2. Type 2 Gumbel Weibull–Uniform (T2GWU) Distribution

Let the baseline distribution H(x,ψ) be a uniform distribution with parameter γ>0. Then, $h\left(x,\gamma \right)=\frac{1}{\gamma }$ and $H\left(x,\gamma \right)=\frac{x}{\gamma }$.

#### 5.2.1. cdf and pdf of the T2GWU Distribution

The cdf of the T2GWU distribution is

(53) $$\begin{array}{c} F_{T2GWU}=\exp\left\{-\alpha {\left(\frac{x}{\gamma -x}\right)}^{-\beta }\right\}, \end{array}$$

and the corresponding pdf is

(54) $$\begin{array}{c} f_{T2GWU}\left(x\right)=\alpha \beta \frac{\gamma x^{-\beta -1}}{{\left(\gamma -x\right)}^{-\beta +1}}\exp\left\{-\alpha {\left(\frac{x}{\gamma -x}\right)}^{-\beta }\right\}. \end{array}$$

#### 5.2.2. Hazard Rate and Quantile Functions

The hrf of T2GWU is displayed by

(55) $$\begin{array}{c} h_{T2GWU}=\frac{\alpha \beta \frac{\gamma x^{-\beta -1}}{{\left(\gamma -x\right)}^{-\beta +1}}\exp\left\{-\alpha {\left(\frac{x}{\gamma -x}\right)}^{-\beta }\right\}}{1-\exp\left\{-\alpha {\left(\frac{x}{\gamma -x}\right)}^{-\beta }\right\}}, \end{array}$$

and the reverse hrf is given by

(56) $$\begin{array}{c} \tau_{T2GWU}=\alpha \beta \frac{\gamma x^{-\beta -1}}{{\left(\gamma -x\right)}^{-\beta +1}}. \end{array}$$

Moreover, the quantile function can be obtained as

(57) $$\begin{array}{c} x_{p}=\frac{1}{1+{\left[\frac{\log p}{-\alpha }\right]}^{\frac{1}{\beta }}}. \end{array}$$

**Figure 3:**
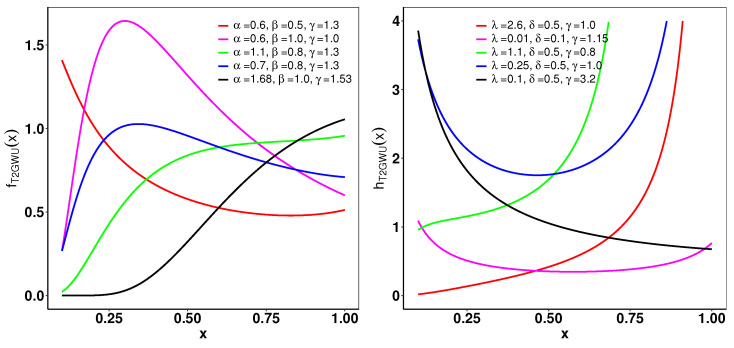
(**left**): Pdf of T2GWU distribution for different values of parameters α, β, and γ. (**right**): Hrf of T2GWU for selected parameters α, β, and γ.

Figure 3 shows the plots of the pdf and hrf for the T2GWU distribution with several combinations of parameter values. The pdf plots show different shapes, including right-skewed, decreasing, and increasing. In addition, the hrf plots capture various possibilities such as increasing, decreasing, bathtub, and shallow bathtub.

### 5.3. Type 2 Gumbel Weibull–Pareto (T2GWP) Distribution

If we set H(x,ψ) as a Pareto distribution with parameter θ,k>0, then $h\left(x,\theta ,k\right)=\frac{k\theta^{k}}{x^{k+1}}$ and $H\left(x,\theta ,k\right)=1-{\left(\frac{\theta }{x}\right)}^{k}$.

#### 5.3.1. cdf and pdf of the T2GWP Distribution

Thus, the cdf of the T2GWP distribution is given by

(58) $$\begin{array}{c} F_{T2GWP}=\exp\left\{-\alpha {\left({\left[\frac{x}{\theta }\right]}^{k}-1\right)}^{-\beta }\right\}, \end{array}$$

with a pdf

(59) $$\begin{array}{c} f_{T2GWP}\left(x\right)=\beta \alpha kx^{k-1}\theta^{-k}{\left({\left[\frac{x}{\theta }\right]}^{k}-1\right)}^{-\beta -1}\exp\left\{-\alpha {\left({\left[\frac{x}{\theta }\right]}^{k}-1\right)}^{-\beta }\right\}. \end{array}$$

#### 5.3.2. Hazard Rate and Quantile Functions

The hrf of T2GWP is given by

(60) $$\begin{array}{c} h_{T2GWP}=\frac{\beta \alpha kx^{k-1}\theta^{-k}{\left({\left[\frac{x}{\theta }\right]}^{k}-1\right)}^{-\beta -1}\exp\left\{-\alpha {\left({\left[\frac{x}{\theta }\right]}^{k}-1\right)}^{-\beta }\right\}}{1-\exp\left\{-\alpha {\left({\left[\frac{x}{\theta }\right]}^{k}-1\right)}^{-\beta }\right\}}, \end{array}$$

and the reverse hrf is

(61) $$\begin{array}{c} \tau_{T2GWP}=\beta \delta \alpha^{-\delta }kx^{k-1}\theta^{-k}{\left({\left[\frac{x}{\theta }\right]}^{k}-1\right)}^{-\beta \delta -1}. \end{array}$$

Moreover, the quantile function is obtained as

(62) $$\begin{array}{c} x_{p}=\theta {\left({\left[\frac{\log p}{-\alpha }\right]}^{-\frac{1}{\beta }}+1\right)}^{\frac{1}{k}} \end{array}$$

**Figure 4:**
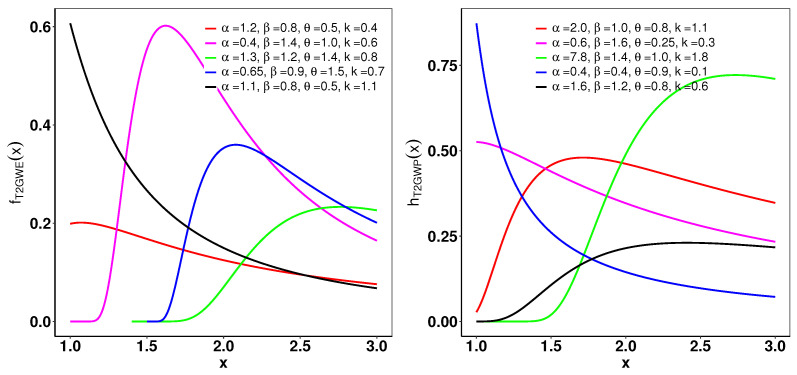
(**left**): The pdf of the T2GWP distribution for selected values of α, β, θ, and *k*. (**right**): The hrf of the T2GWP for various α, β, θ, and *k*.

Shapes of the pdf and hrf for the T2GWP distribution with selected parameters are shown in Figure 4. The pdfs exhibit a variety of shapes, including right-skewed, decreasing, and increasing. Moreover, hrf plots for the T2GWP distribution display growing, decreasing, and right-skewed forms.

## 6. Applications

In this section, we will investigate beyond theoretical constructs and delve into the practical implications of our model, demonstrating its applicability using real-world datasets. This will validate the practical utility of our newly devised model and shed light on how it can be effectively employed in handling concrete data-driven scenarios. The objective is to ensure that our theoretical advancements resonate with tangible applications, thereby significantly contributing to both the academic discourse and the operational applications of statistical distributions.

In this section, we present three applications of the Type 2 Gumbel Weibull–uniform and exponential distribution. We compared it with the Exponentiated Gumbel Type 2 (EGT) [37], Weibull Generalized Exponential (WGE) [38], Lomax Gumbel Type 2 (LGT) [39], Type 2 Gumbel (T2G), and gamma distribution (GM) and Weibull distribution (WB). The pdf and cdf of those distributions are provided in Appendix B. The goodness-of-fit statistics including the -2log-likelihood statistic, Cramér–von Mises statistic ($W^{*}$), Anderson–Darling statistic ($A^{*}$), Akaike Information Criterion (AIC), Bayesian Information Criterion (BIC), Consistent Akaike Information Criterion (CAIC), Hannan–Quinn criterion (HQIC), and Kolmogorov–Smirnov test statistic (K-S) and its corresponding *p*-value are reported.

To evaluate and compare the performance of different models, we can examine their goodness-of-fit statistics. Generally, a model with smaller values in these statistics fits the data better. However, it is important to note that, for the *p*-values, which are a measure of expectation, a larger value indicates a better fit. To access the data discussed in this section, please refer to the “Declarations” section for guidance.

### 6.1. Aarset Data

**Figure 5:**
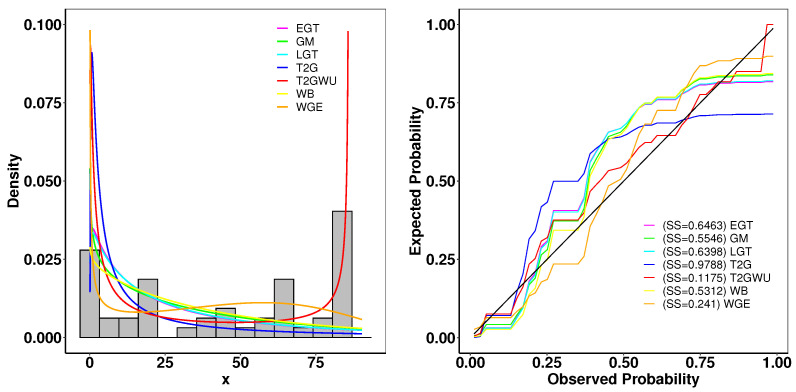
(**left**): Fitted density superposed on the histogram and observed probability for the Aarset data. (**right**): Expected probability plots for the Aarset data.

**Figure 6:**
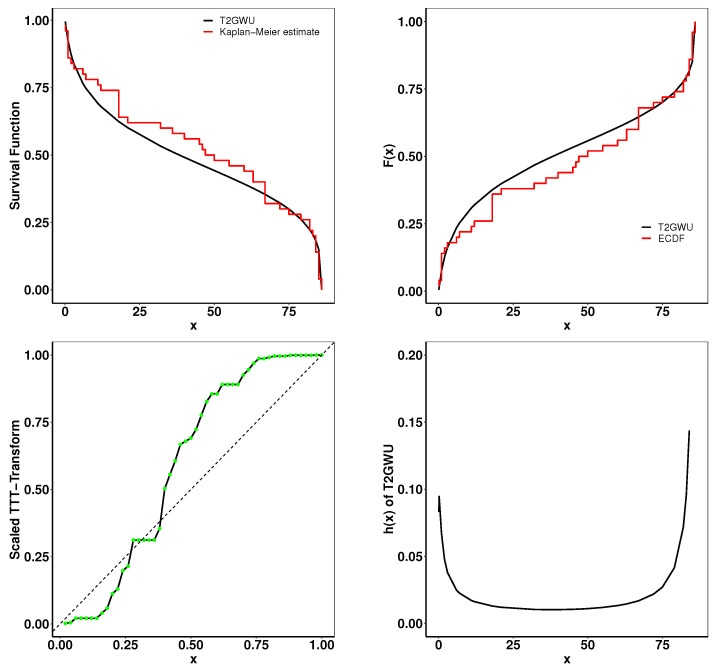
Fitted K-M survival curve, theoretical and empirical cdfs, the TTT statistics, and the hrf for the Aarset data.

This Aarset dataset recorded the lifetimes of 50 devices [40]. The estimated parameters and the goodness-of-fit statistics are presented in Table 4. Figure 5 presents the plots of the fitted densities alongside the histogram and the expected probability. In Figure 6, the Kaplan–Meier (K–M) survival curve, the theoretical cumulative distribution function and empirical cumulative distribution function (ECDF), and total time on test (TTT) scaled are displayed. The closely matched empirical and theoretical plots suggest that our model is an excellent fit for the given data. Additionally, the TTT scaled plot shows that the model is suitable for a hazard rate structure that is not monotonic.

### 6.2. Meeker and Escobar Data

**Figure 7:**
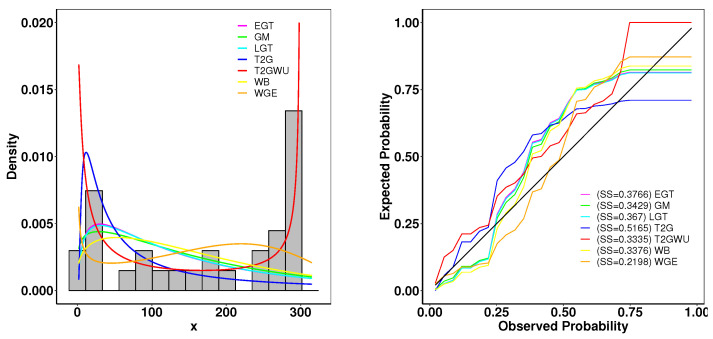
(**left**): Fitted density superposed on the histogram and observed probability for the Aarset data. (**right**): Expected probability plots for the Meeker and Escobar data.

**Figure 8:**
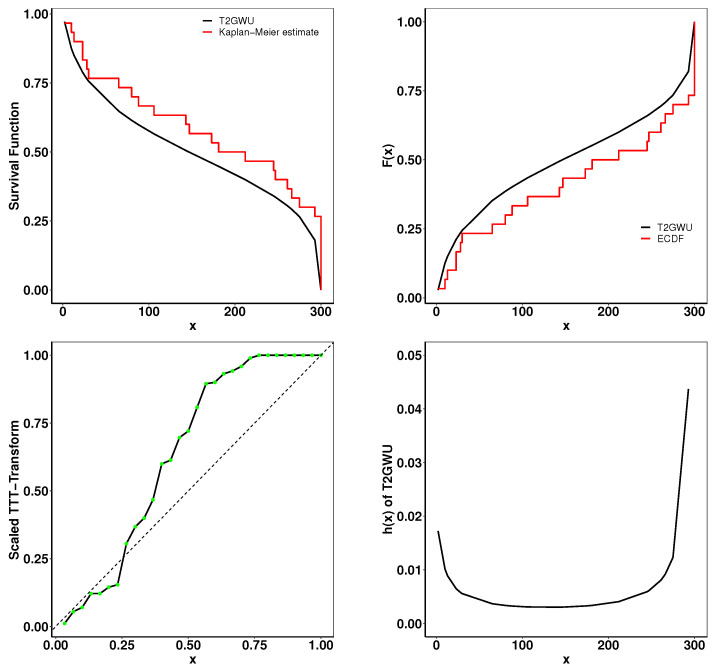
Fitted K-M survival curve, theoretical and empirical cdf, the TTT statistics, and the hrf for the Meeker and Escobar data.

This dataset consists of the running times of 30 devices. From Table 5 and Figure 7, we can compare the Type 2 Gumbel Weibull–uniform distribution with other distributions for various goodness-of-fit methods. As shown in Figure 8, the close resemblance between the fitted empirical and theoretical plots indicates a strong fit of our model to the provided data. Furthermore, the TTT scaled plot provides clear evidence that the model is appropriate for a non-monotonic hazard rate structure.

### 6.3. Chemotherapy Data

**Figure 9:**
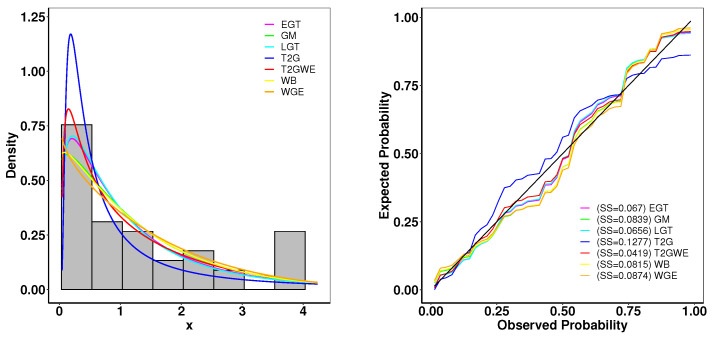
(**left**): Fitted density superposed on the histogram and observed probability for the Chemotherapy data. (**right**): Expected probability plots for the Chemotherapy data.

This dataset is a subset of data reported by Bekker et al. [41], which represents the survival times (in years) of a group of 45 patients who received chemotherapy treatment alone. The estimates of the parameters and the goodness-of-fit statistics are summarized in Table 6. Figure 9 plots the fitted densities alongside the histogram and the expected probability.

**Figure 10:**
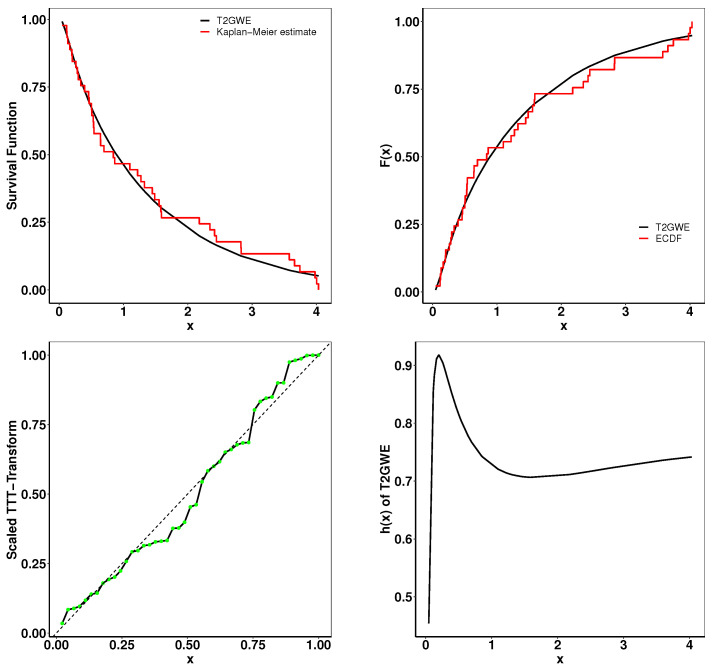
Fitted K-M survival curve, theoretical and empirical cdfs, the TTT statistics, and the hrf for the Chemotherapy data.

In Figure 10, the K–M survival curve, as well as the theoretical and empirical cdfs, and TTT scaled are displayed.

The above examples evaluate the performance and flexibility of the proposed Type 2 Gumbel Weibull-G family of distributions using three distinct datasets: the Aarset data, Meeker and Escobar data, and Chemotherapy data. The findings are summarized as follows:

1. **Goodness-of-Fit Analysis:** Across all datasets, the proposed distributions demonstrate superior performance compared to existing models. This is evidenced by their lower goodness-of-fit statistics, including -2log-likelihood statistic, $W^{*}$, $A^{*}$, AIC, BIC, CAIC, HQIC, and K-S and its higher *p*-values. These metrics confirm the robustness of the proposed distributions in capturing complex data patterns.
2. **Empirical and Theoretical Fits:** The fitted densities closely resemble the empirical histograms and expected probability plots, indicating a strong fit. Additionally, survival curves (Kaplan–Meier estimates) and cumulative distribution functions align well with the theoretical models, supporting the applicability of the proposed family to survival analysis.
3. **Hazard Rate Characteristics:** The total time on test (TTT) and hazard rate plots highlight the capability of the proposed distributions to model non-monotonic hazard rate structures, such as bathtub-shaped curves, which are often challenging for traditional distributions.

## 7. Conclusions

This paper introduces a novel methodology for generating continuous statistical distributions by utilizing the exponentiated odds ratio, which is based on the concepts of survival analysis. The approach described herein constitutes a substantial advancement in the field of statistical modeling, effectively augmenting the flexibility and accuracy of distribution models to effectively address the requirements posed by intricate contemporary data architectures. The major point of this progress lies in the formulation of the “Type 2 Gumbel Weibull-G family of distributions”, which has undergone a comprehensive mathematical analysis. The scope of this investigation covered various statistical properties, such as expansions of density functions, moments, hazard rate and quantile functions, Rényi entropy, order statistics, and an examination of stochastic ordering.

In order to assess the robustness and reliability of the new generator, we employed a set of five advanced parameter estimation techniques: maximum likelihood, least square, weighted least square, maximum product spacing, Cramér–von Mises, and Anderson and Darling. The efficacy and utility of the Type 2 Gumbel Weibull-G distributions were further validated through a comprehensive analysis of three datasets obtained from real-world scenarios. These practical implementations demonstrated the superior statistical accuracy of our proposed distributions over existing models, thereby emphasizing their relevance and applicability in both theoretical and practical statistical domains.

As highlighted in our recent work [28,29], the proposed generator shows significant potential for producing a wider variety of density functions, including more bathtub and complex shapes. Our future research endeavors will entail a thorough investigation of several more sub-families within the new generator. This exploration will focus on their distinct properties and potential applications in diverse scenarios, setting them in comparison with other established distribution models. Furthermore, we are now developing an R package (version 4.4.2) with the objective of optimizing the parameter estimate process through the utilization of diverse methodologies. This will result in the improved efficiency of data-fitting procedures, thus increasing the accessibility and practicality of our study for wider applications.

## Figures and Tables

**Table 1:** Families of distributions derived from different R(t).

Distribution	R(t)	RT-EOR-H
Uniform	$\frac{t}{\theta }$	$\frac{\alpha }{\theta }{\left[\frac{H(x,\psi )}{\bar{H}\left(x,\psi \right)}\right]}^{\beta }$
Normal	$\Phi \left(\frac{x-\mu }{\sigma }\right)$	$\Phi \left(\frac{\alpha {\left[\frac{H(x,\psi )}{\bar{H}\left(x,\psi \right)}\right]}^{\beta }-\mu }{\sigma }\right)$
Gamma	$\frac{1}{\Gamma (k)}\gamma \left(k,\frac{t}{\theta }\right)$	$\frac{1}{\Gamma (k)}\gamma \left(k,\frac{\alpha }{\theta }{\left[\frac{H(x,\psi )}{\bar{H}\left(x,\psi \right)}\right]}^{\beta }\right)$
Log-logistic	$\frac{1}{1+{\left(t/c\right)}^{-k}}$	$\frac{1}{1+{\left(\frac{\alpha }{c}{\left[\frac{H(x,\psi )}{\bar{H}\left(x,\psi \right)}\right]}^{\beta }\right)}^{-k}}$
Rayleigh	$1-e^{-\frac{t^{2}}{2\sigma^{2}}}$	$1-\exp\left\{-\frac{\alpha^{2}}{2\sigma^{2}}{\left[\frac{H(x,\psi )}{\bar{H}\left(x,\psi \right)}\right]}^{2\beta }\right\}$
Weibull	$1-e^{-{\left(t/\lambda \right)}^{k}}$	$1-\exp\left\{-{\left(\frac{\alpha }{\lambda }{\left[\frac{H(x,\psi )}{\bar{H}\left(x,\psi \right)}\right]}^{\beta }\right)}^{k}\right\}$
Type 2 Gumbel	$e^{-\lambda t^{-\delta }}$	$\exp\left\{-\lambda {\left(\alpha {\left[\frac{H(x,\psi )}{\bar{H}\left(x,\psi \right)}\right]}^{\beta }\right)}^{-\delta }\right\}$
Lomax	$1-{\left(1+\lambda t\right)}^{-k}$	$1-{\left(1+\lambda \alpha {\left[\frac{H(x,\psi )}{\bar{H}\left(x,\psi \right)}\right]}^{\beta }\right)}^{-k}$
Burr XII	$1-{\left(1+t^{c}\right)}^{-k}$	$1-{\left\{1+{\left(\alpha {\left[\frac{H(x,\psi )}{\bar{H}\left(x,\psi \right)}\right]}^{\beta }\right)}^{c}\right\}}^{-k}$
Pareto	$1-{\left(\frac{\gamma }{t}\right)}^{k}$	$1-{\left(\frac{\gamma }{\alpha {\left[\frac{H(x,\psi )}{\bar{H}\left(x,\psi \right)}\right]}^{\beta }}\right)}^{k}$
Lévy	$\mathrm{erfc}\left(\sqrt{\frac{c}{2t}}\right)$	$\mathrm{erfc}\left(\sqrt{\frac{c}{2\alpha {\left[\frac{H(x,\psi )}{\bar{H}\left(x,\psi \right)}\right]}^{\beta }}}\right)$
Fréchet	$e^{-\lambda t^{\gamma }}$	$\exp\left\{-\lambda {\left(\alpha {\left[\frac{H(x,\psi )}{\bar{H}\left(x,\psi \right)}\right]}^{\beta }\right)}^{\gamma }\right\}$
Kumaraswamy	${\left(1-x^{k}\right)}^{-\lambda }-1$	${\left\{1-{\left(\alpha {\left[\frac{H(x,\psi )}{\bar{H}\left(x,\psi \right)}\right]}^{\beta }\right)}^{k}\right\}}^{-\lambda }-1$

**Table 2:** Pseudo-code for Monte Carlo simulation.

Step	Description
**Input**	Select a set of true parameters, for example, α=2.5, β=0.8, γ=1.3. Sample sizes: N={50,100,250,500,1000}. Number of repetitions: R=1000. Initial values for optimization: init_params $=[\alpha_{0},\beta_{0},\gamma_{0}]$.
**Output**	Mean parameter estimates and error metrics for each sample size.
**Step 1**	Define the model-specific function and transformation rules.
**Step 2**	Initialize an empty data structure (e.g., table) to store results.
**Step 3**	For each sample size *n* in *N*:
	Initialize a results container to store parameter estimates and errors. For each replication r=1 to *R*: Generate a random sample of size *n* from the model. Initialize arrays to store intermediate values (e.g., sample data X, estimation errors). For each data point *i* in the sample, – Compute $y_{i}$ using the model with parameters (α,β,γ). – Store $y_{i}$ in the sample array X. – Calculate indices or statistics required for estimation (e.g., MLE, WLS). Sort X in descending order to prepare for estimation. Apply estimation techniques to compute parameter estimates. Record the estimation errors in the results container. Compute mean parameter estimates and error metrics (e.g., MSE) for sample size *n*.
**Step 4**	Aggregate results for all sample sizes.
**Return**	Summary of mean parameter estimates and error metrics.

**Table 3:** Different estimations for α=2.5,β=0.8,γ=1.3.

		MLE		LS		WLS		MPS		CVM		AD	
N		Bias	MSE	Bias	MSE	Bias	MSE	Bias	MSE	Bias	MSE	Bias	MSE
	α	0.0909	2.0761	−0.0418	2.9292	0.0307	2.6645	0.8349	2.8174	0.1095	3.2913	0.1459	2.5743
50	β	0.0512	0.0778	0.0107	0.1906	0.0066	0.1520	−0.1492	0.1068	0.0262	0.1929	0.0010	0.1192
	γ	0.1512	0.8690	0.9373	8.7264	0.8477	9.4008	2.4409	63.8413	0.8518	6.9832	0.6359	5.4760
	α	0.0662	1.1061	0.0595	1.6917	0.0878	1.4001	0.5914	1.5128	0.1357	1.7947	0.1327	1.3275
100	β	0.0182	0.0379	−0.0229	0.0897	−0.0158	0.0586	−0.1047	0.0511	−0.0161	0.0895	−0.0163	0.0520
	γ	0.0963	0.3519	0.4901	3.1145	0.2684	1.1972	0.8138	14.1750	0.4755	2.7094	0.2518	0.9787
	α	0.0203	0.4578	0.0211	0.8317	0.0336	0.5983	0.3204	0.5872	0.0510	0.8502	0.0533	0.5754
250	β	0.0097	0.0148	−0.0027	0.0349	−0.0010	0.0211	−0.0544	0.0177	−0.0002	0.0350	−0.0027	0.0197
	γ	0.0265	0.1085	0.1088	0.3554	0.0614	0.1707	0.2180	0.2020	0.1141	0.3529	0.0660	0.1620
	α	0.0226	0.2394	0.0168	0.4176	0.0220	0.2933	0.2066	0.2890	0.0318	0.4223	0.0321	0.2900
500	β	0.0049	0.0075	−0.0015	0.0168	0.0002	0.0102	−0.0335	0.0086	−0.0003	0.0168	−0.0008	0.0100
	γ	0.0189	0.0543	0.0498	0.1280	0.0307	0.0742	0.1269	0.0809	0.0529	0.1282	0.0347	0.0744
	α	0.0072	0.1146	0.0071	0.2278	0.0113	0.1497	0.1163	0.1310	0.0146	0.2290	0.0167	0.1504
1000	β	0.0027	0.0036	−0.0003	0.0086	0.0001	0.0050	−0.0198	0.0039	0.0003	0.0086	−0.0007	0.0050
	γ	0.0079	0.0254	0.0258	0.0673	0.0160	0.0373	0.0691	0.0331	0.0274	0.0673	0.0186	0.0380

**Table 4:** MLEs and goodness-of-fit statistics for Aarset data.

	Estimates (SE)				Statistics								
**Model**	α	β	γ		−2logL	AIC	CAIC	BIC	HQIC	$W^{*}$	$A^{*}$	K-S	*p*-Value
T2GWU	0.6470	0.3112	86.0000	-	361.1643	367.1643	371.0961	372.9003	369.3486	0.3358	2.2924	0.1604	0.1524
	α	ϕ	θ										
EGT	1000.1042	0.1088	10.3528	-	492.5518	498.5519	499.0736	504.2880	500.7362	0.6258	3.6601	0.2164	0.0185
	($1.0496\times 10^{3}$)	($1.7639\times 10^{-2}$)	(1.1897)										
	α	θ	γ										
WGE	0.1742	0.3851	0.0778	-	451.2370	457.2371	457.7588	462.9731	459.4214	0.2120	1.4796	0.1288	0.3778
	(0.0620)	(0.1028)	(0.0257)										
	α	β	θ	*k*									
LGT	31.9188	0.0093	11.6179	0.0959	491.9342	499.9343	500.8232	507.5824	502.8467	0.6163	3.6145	0.2160	0.0189
	(30.3417)	(0.0061)	(1.2409)	(0.0154)									
	α	ν											
T2G	2.6477	0.4633	-	-	530.0282	534.0281	534.2835	537.8522	535.4844	1.0407	5.5694	0.2855	0.0006
	(0.3898)	(0.0444)											
	*k*	λ											
WB	0.9488	44.8440	-	-	482.0038	486.0037	486.2591	489.8278	487.4600	0.4964	3.0078	0.1933	0.0476
	(0.1195)	(6.9313)											
	α	β											
GM	0.7995	0.0175	-	-	480.3804	484.3804	484.6358	488.2045	485.8367	0.4892	2.9700	0.2022	0.0335
	(0.1376)	(0.0041)											

**Table 5:** MLEs and goodness-of-fit statistics for Meeker and Escobar data.

	Estimates (SE)				Statistics								
**Model**	α	β	γ		−2logL	AIC	CAIC	BIC	HQIC	$W^{*}$	$A^{*}$	K-S	*p*-Value
T2GWU	0.6825	0.3305	300.00	-	86.4070	92.4070	95.8410	96.6106	93.7518	0.2427	1.6235	0.1894	0.2319
	(8.8328)	(0.2076)	(0.2675)										
	α	ϕ	θ										
EGT	1000.6472	0.1470	14.7781	-	374.4318	380.4317	381.3548	384.6353	381.7765	0.3617	2.0879	0.2170	0.1186
	($1.3869\times 10^{3}$)	($3.1342\times 10^{-2}$)	(2.0011)										
	α	θ	γ										
WGE	0.1253	0.4986	0.0187	-	354.3142	360.3143	361.2374	364.5179	361.659	0.1938	1.3025	0.1728	0.3322
	(0.0676)	(0.2096)	(0.0097)										
	α	β	θ	*k*									
LGT	20.7564	0.0065	15.7890	0.1303	374.4282	382.4283	384.0283	388.0331	384.2213	0.3612	2.0865	0.2143	0.1272
	(19.6057)	(0.0040)	(1.8170)	(0.0240)									
	α	ν											
T2G	12.1053	0.6252	-	-	396.8938	400.8938	401.3383	403.6962	401.7903	0.5541	3.0254	0.2898	0.0129
	(3.6124)	(0.0761)											
	α	β											
WB	1.2626	186.8126	-	-	368.6296	372.6296	373.0741	375.4320	373.5261	0.3035	1.8207	0.2221	0.1037
	(0.2042)	(27.9960)											
	α	β											
GM	1.1931	0.0067	-	-	370.0416	374.0415	374.4860	376.8439	374.938	0.3212	1.9044	0.2172	0.1179
	(0.2677)	(0.0018)											

**Table 6:** MLEs and goodness-of-fit statistics for Chemotherapy data.

	Estimates (SE)				Statistics								
**Model**	α	β	γ		−2logL	AIC	CAIC	BIC	HQIC	$W^{*}$	$A^{*}$	K-S	*p*-Value
T2GWE	1.1328	0.5416	1.4015	-	113.3334	119.3334	119.9188	124.7534	121.3539	0.0415	0.3113	0.0756	0.9421
	(0.4388)	(0.1170)	(0.5530)										
	α	ϕ	θ										
EGT	1000.1282	0.1452	7.1554	-	115.9096	121.9096	122.4949	127.3295	123.9301	0.0608	0.4231	0.0927	0.0926
	($3.6913\times 10^{3}$)	($7.4598\times 10^{-2}$)	(3.7373)										
	α	θ	γ										
WGE	3.9393	0.9508	0.1484	-	115.9251	121.9251	122.5105	127.3451	123.9457	0.0917	0.6079	0.1120	0.5864
	(8.8328)	(0.2076)	(0.2675)										
	α	β	θ	*k*									
LGT	17.9903	0.0196	7.0332	0.1503	116.3564	124.3564	125.3564	131.5831	127.0504	0.0610	0.4270	0.0892	0.835
	(24.8990)	(0.0298)	(1.9777)	(0.0459)									
	α	ν											
T2G	0.4987	0.8672	-	-	127.6381	131.6381	131.9238	135.2515	132.9851	0.1430	0.9790	0.1382	0.3253
	(0.0979)	(0.0928)											
	*k*	λ											
WB	1.0532	1.3700	-	-	116.2474	120.2474	120.5331	123.8608	121.5944	0.0813	0.5436	0.1094	0.6146
	(0.1238)	(0.2048)											
	α	β											
GM	1.1007	0.8205	-	-	480.3804	120.1815	120.4672	123.7948	121.5285	0.0790	0.5295	0.1106	0.6016
	(0.2060)	(0.1928)											

## Data Availability

All data utilized in this study are openly available on GitHub. The dataset can be accessed through the provided link: https://github.com/shusenpu/Generator_Data, accessed on 15 October 2024. The data can also be accessed using their Digital Object Identifier (DOI): 10.5281/zenodo.10215787. To retrieve the data, visit the following link: https://doi.org/10.5281/zenodo.10215787.

## Abbreviations

The following abbreviations are used in this manuscript:

T2GWG	The Type 2 Gumbel Weibull-G
cdf	cumulative distribution function
pdf	probability density function
hrf	hazard rate function
Exp-G	exponentiated-G
EGT	Exponentiated Gumbel Type 2
WGE	Weibull Generalized Exponential
LGT	Lomax Gumbel Type 2
T2G	Type 2 Gumbel
EWL	Exponentiated Weibull–logistic distribution
MLE	maximum likelihood estimate
MPS	maximum product spacing estimate
LS	least square estimate
WLS	weighted least square estimate
CVM	Cramér–von Mises estimate
AD	Anderson and Darling estimate
T2GWE	Type 2 Gumbel Weibull–exponential
T2GWU	Type 2 Gumbel Weibull–uniform
T2GWP	Type 2 Gumbel Weibull–Pareto
AIC	Akaike Information Criterion
CAIC	Consistent Akaike Information Criterion
BIC	Bayesian Information Criterion
HQIC	Hannan–Quinn Criterion
$W^{*}$	Cramér–von Mises statistic
$A^{*}$	Anderson–Darling statistic
K-S	Kolmogorov–Smirnov statistic
ECDF	empirical cumulative distribution function
TTT	total time on test
K-M	Kaplan–Meier

## Appendix A. The First Derivatives of H

The first partial derivatives of *H* in Section 4 with respect to $\alpha ,\beta ,\delta ,\psi_{k}$ are given by

(A1) $$\begin{array}{cc} \frac{\partial W}{\partial \alpha }= & \frac{1}{n+1}\left\{-{\left[\frac{H(x_{1},\psi )}{\bar{H}\left(x_{1},\psi \right)}\right]}^{-\beta }+\frac{{\left[\frac{H(x_{n},\psi )}{\bar{H}\left(x_{n},\psi \right)}\right]}^{-\beta }\exp\left\{-\alpha {\left[\frac{H(x_{i},\psi )}{\bar{H}\left(x_{i},\psi \right)}\right]}^{-\beta }\right\}}{1-\exp\left\{-\alpha {\left[\frac{H(x_{i},\psi )}{\bar{H}\left(x_{i},\psi \right)}\right]}^{-\beta }\right\}}\right.\\ \; & +\sum_{i=2}^{n}\left[\frac{{\left[\frac{H(x_{i},\psi )}{\bar{H}\left(x_{i},\psi \right)}\right]}^{-\beta }\exp\left\{-\alpha {\left[\frac{H(x_{i},\psi )}{\bar{H}\left(x_{i},\psi \right)}\right]}^{-\beta }\right\}}{\exp\left\{-\alpha {\left[\frac{H(x_{i},\psi )}{\bar{H}\left(x_{i},\psi \right)}\right]}^{-\beta }\right\}-\exp\left\{-\alpha {\left[\frac{H(x_{i-1},\psi )}{\bar{H}\left(x_{i-1},\psi \right)}\right]}^{-\beta }\right\}}\right.\\ \; & \left.\left.-\frac{{\left[\frac{H(x_{i-1},\psi )}{\bar{H}\left(x_{i-1},\psi \right)}\right]}^{-\beta }\exp\left\{-\alpha {\left[\frac{H(x_{i-1},\psi )}{\bar{H}\left(x_{i-1},\psi \right)}\right]}^{-\beta }\right\}}{\exp\left\{-\alpha {\left[\frac{H(x_{i},\psi )}{\bar{H}\left(x_{i},\psi \right)}\right]}^{-\beta }\right\}-\exp\left\{-\alpha {\left[\frac{H(x_{i-1},\psi )}{\bar{H}\left(x_{i-1},\psi \right)}\right]}^{-\beta }\right\}}\right]\right\} \end{array}$$

(A2) $$\begin{array}{cc} \frac{\partial W}{\partial \beta }= & \frac{1}{n+1}\left\{\alpha \log\left(\frac{H(x_{1},\psi )}{\bar{H}\left(x_{1},\psi \right)}\right){\left[\frac{H(x_{i},\psi )}{\bar{H}\left(x_{i},\psi \right)}\right]}^{-\beta }\right.\\ \; & +\frac{\log\left(\frac{H(x_{n},\psi )}{\bar{H}\left(x_{n},\psi \right)}\right){\left[\frac{H(x_{n},\psi )}{\bar{H}\left(x_{n},\psi \right)}\right]}^{-\beta }\exp\left\{-\alpha {\left[\frac{H(x_{i},\psi )}{\bar{H}\left(x_{i},\psi \right)}\right]}^{-\beta }\right\}}{1-\exp\left\{-\alpha {\left[\frac{H(x_{i},\psi )}{\bar{H}\left(x_{i},\psi \right)}\right]}^{-\beta }\right\}}\\ \; & +\sum_{i=2}^{n}\left[\frac{\log\left(\frac{H(x_{i},\psi )}{\bar{H}\left(x_{i},\psi \right)}\right){\left[\frac{H(x_{i},\psi )}{\bar{H}\left(x_{i},\psi \right)}\right]}^{-\beta }\exp\left\{-\alpha {\left[\frac{H(x_{i},\psi )}{\bar{H}\left(x_{i},\psi \right)}\right]}^{-\beta }\right\}}{\exp\left\{-\alpha {\left[\frac{H(x_{i},\psi )}{\bar{H}\left(x_{i},\psi \right)}\right]}^{-\beta }\right\}-\exp\left\{-\alpha {\left[\frac{H(x_{i-1},\psi )}{\bar{H}\left(x_{i-1},\psi \right)}\right]}^{-\beta }\right\}}\right.\\ \; & \left.\left.\left.-\frac{\log\left(\frac{H(x_{i-1},\psi )}{\bar{H}\left(x_{i-1},\psi \right)}\right){\left[\frac{H(x_{i-1},\psi )}{\bar{H}\left(x_{i-1},\psi \right)}\right]}^{-\beta }\exp\left\{-\alpha {\left[\frac{H(x_{i-1},\psi )}{\bar{H}\left(x_{i-1},\psi \right)}\right]}^{-\beta }\right\}}{\exp\left\{-\alpha {\left[\frac{H(x_{i},\psi )}{\bar{H}\left(x_{i},\psi \right)}\right]}^{-\beta }\right\}-\exp\left\{-\alpha {\left[\frac{H(x_{i-1},\psi )}{\bar{H}\left(x_{i-1},\psi \right)}\right]}^{-\beta }\right\}}\right]\right)\right\} \end{array}$$

(A3) $$\begin{array}{cc} \frac{\partial W}{\partial \psi_{k}}= & \frac{1}{n+1}\left\{\alpha \beta \frac{H{\left(x_{1},\psi \right)}^{-\beta -1}}{\bar{H}{\left(x_{1},\psi \right)}^{-\beta +1}}\frac{\partial H(x_{1},\psi )}{\partial \psi_{k}}+\frac{\frac{H{\left(x_{n},\psi \right)}^{-\beta -1}}{\bar{H}{\left(x_{n},\psi \right)}^{-\beta +1}}\frac{\partial H(x_{n},\psi )}{\partial \psi_{k}}\exp\left\{-\alpha {\left[\frac{H(x_{n},\psi )}{\bar{H}\left(x_{n},\psi \right)}\right]}^{-\beta }\right\}}{1-\exp\left\{-\alpha {\left[\frac{H(x_{n},\psi )}{\bar{H}\left(x_{n},\psi \right)}\right]}^{-\beta }\right\}}\right.\\ \; & +\sum_{i=2}^{n}\left[\frac{\frac{H{\left(x_{i},\psi \right)}^{-\beta -1}}{\bar{H}{\left(x_{i},\psi \right)}^{-\beta +1}}\frac{\partial H(x_{i},\psi )}{\partial \psi_{k}}\exp\left\{-\alpha {\left[\frac{H(x_{i},\psi )}{\bar{H}\left(x_{i},\psi \right)}\right]}^{-\beta }\right\}}{\exp\left\{-\alpha {\left[\frac{H(x_{i},\psi )}{\bar{H}\left(x_{i},\psi \right)}\right]}^{-\beta }\right\}-\exp\left\{-\alpha {\left[\frac{H(x_{i-1},\psi )}{\bar{H}\left(x_{i-1},\psi \right)}\right]}^{-\beta }\right\}}\right.\\ \; & \left.\left.\left.-\frac{\frac{H{\left(x_{i-1},\psi \right)}^{-\beta -1}}{\bar{H}{\left(x_{i-1},\psi \right)}^{-\beta +1}}\frac{\partial H(x_{i-1},\psi )}{\partial \psi_{k}}\exp\left\{-\alpha {\left[\frac{H(x_{i-1},\psi )}{\bar{H}\left(x_{i-1},\psi \right)}\right]}^{-\beta }\right\}}{\exp\left\{-\alpha {\left[\frac{H(x_{i},\psi )}{\bar{H}\left(x_{i},\psi \right)}\right]}^{-\beta }\right\}-\exp\left\{-\alpha {\left[\frac{H(x_{i-1},\psi )}{\bar{H}\left(x_{i-1},\psi \right)}\right]}^{-\beta }\right\}}\right]\right)\right\} \end{array}$$

## Appendix B. Distributions Used in the Application Section

- Exponentiated Gumbel Type 2 Distribution:
  $$F_{EGT}\left(x\right)=1-{\left(1-e^{-\theta x^{-\phi }}\right)}^{\alpha },$$
  $$f_{EGT}\left(x\right)=\alpha \phi \theta x^{-\phi -1}e^{-\theta x^{-\phi }}{\left(1-e^{-\theta x^{-\phi }}\right)}^{\alpha -1},$$
  α,ϕ,θ>0
- Weibull Generalized Exponential Distribution:
  $$F_{WGE}\left(x\right)=1-e^{-a{\left[e^{\lambda x}-1\right]}^{b}},$$
  $$f_{WGE}\left(x\right)=ab\lambda e^{\lambda x}{\left[e^{\lambda x}-1\right]}^{b-1}e^{-a{\left[e^{\lambda x}-1\right]}^{b}},$$
  a,b,λ>0
- Lomax Gumbel Type 2 Distribution:
  $$F_{LGT}\left(x\right)=1-\beta^{\alpha }{\left(\beta -\log\left[1-e^{-\theta x^{-k}}\right]\right)}^{-\alpha }$$
  $$f_{LGT}\left(x\right)=\frac{\alpha \beta^{\alpha }\theta kx^{-k-1}e^{-\theta x^{-k}}}{\left[1-e^{-\theta x^{-k}}\right]}{\left[\beta -\log\left[1-e^{-\theta x^{-k}}\right]\right]}^{-(\alpha +1)}$$
  α,β,θ,k>0
- Type 2 Gumbel Distribution:
  $$F_{T2G}\left(x\right)=e^{-\theta x^{-\phi }}$$
  $$f_{T2G}\left(x\right)=\phi \theta x^{-\phi -1}e^{-\theta x^{-\phi }}$$
  ϕ,θ>0
- Exponentiated Weibull–Logistic Distribution:
  $$F_{EWL}\left(x\right)={\left(1-e^{-\alpha e^{\lambda \beta x}}\right)}^{\theta }$$
  $$f_{EWL}\left(x\right)=\theta {\left(1-e^{-\alpha e^{\lambda \beta x}}\right)}^{\theta -1}\left(\lambda \alpha \beta e^{\lambda \beta x-\alpha e^{\lambda \beta x}}\right),$$
  λ,α,β,θ>0.
